# Acute Pulmonary Edema Due to Excessive Water Intake in Pyschiatric Patient

**DOI:** 10.5812/ircmj.2228

**Published:** 2013-04-05

**Authors:** Hüseyin Narcı

**Affiliations:** 1 Baskent University, Emergency Department, Konya, Turkey

**Keywords:** Water Intoxication, Pulmonary Edema, Patients

## Dear Editor,

Water taking diseases are frequent in psychiatric patient. Approximately 3-6% of psychiatric patient water intoxication is seen. Although water intoxication is defined well, the metabolism of this is not understood. In psychiatric patients, headache, ataxia, confusion, urinary incontinence and electrolyte imbalance is reported. If not treated, confusion, acute delirium, coma and death is seen. Cerebellar edema, pulmonary edema, electrolyte imbalance and urinary incontinence due to water intoxication are reported (1). In this case diagnosis and treatment of psychiatric patient that take excessive water is discussed. The patient a 50-year-old woman schizophrenic had been drinking large amounts of water for several hours before and rapid onset respiratory distress and confusion. In history she started to have fear and stress before 6 years. These symptoms began to increase progressively and last year agitation also seen. Respiratory distress began due to water intake above 10 liters. She was confused, dispneic on admission. TA: 160/90 mmHg, pulse rate 90, respiration 26/min.

Cardiovascular and neurologic system was normal and rales were heard in both lungs. In laboratory: Glucose: 100, BUN: 7, Creatinine: 0.8, Na: 129, CI: 87, K: 4.2, albumin:4.1, WBC:8000, Hb:12.2, platelet:367000. Blood gas analysis: Ph 7.32, Pco2 55 mmHg, po2 134 mmHg, Bicarbonate; 36.1 and O2Sat: 99.4. His chest X-ray showed bilateral patch consolidation of the lung fields, cardiomegaly was not seen. Echocardiography; ejection fraction 60 % and no failure. These were suggesting non –cardiogenic pulmonary edema due to excessive water intake and treatment started in our emergency department. 5-ml/min oxygen was given, 3 mg intravenous morphine was done and 5-mcg/kg/min nitroglycerin infusion started, 400 mg intravenous furosemide in 12 hours was given. Patient urinate 3500 cc totally in 12 hours.The clinical outcome was favourable after the treatment. She became alert and ralles decreased. After 24 hours all vital symptoms became stable and patient discharged.

Water intoxication is reported in marathon runners, psychiatric patients and soldiers. Psychos, psycotrop drugs, diuretics, nicotine and alcohol can cause excessive water intake. Pyschogenic water intoxication is discussed in the light of 150 observations published in the literature since 1935. 87% of all patients were schizophrenic, and 13% had other psychoses (2). In a long-term psychiatric setting, self-induced water intoxication may be a life-threatening situation. At first glance, the symptoms or behaviors of self-induced water intoxication are similar to schizophrenia, i.e., inappropriate behavior, delusions, hallucinations, confusion, and disorientation.. Affected individuals develop polydipsia, which is accompanied by overhydration and dilutional hyponatremia. If untreated, the symptoms may progress from mild confusion to acute delirium, seizures, coma, or death (3). In our case excessive water intake was due to schizophrenic condition.

Pulmonary edema is a rare complication of water intoxication in a psychiatric patient.The physiology of pulmonary edema caused by excessive water intake can’t be explained. Furosemide and water restriction in these patients give good response (2). In our case, echo was normal, bilateral rales were heard and bilateral opacity was seen, these suggested is non –cardiogenic pulmonary edema. After fluid restriction and intravenous infusion of nitroglyserin and furosemide, she passed large amount of urine, and her consciousness level, and respiratory distres, improved gradually.

Water intoxication provokes disturbances in electrolyte balance, resulting in a rapid decrease in serum sodium concentration and eventual death. Symptoms can become apparent when the serum sodium falls below 120 mmol/litre, but are usually associated with concentrations below 110 mmol/litre. Severe symptoms occur with very low sodium concentrations of 90–105 mmol/litre (4). It is reported that approximately 10-15 liters day water intake by an psychiatric patient is caused to consciousness (1). In our case, water intake was 10 liters and patient was confused and Na level of 129. In emergency department, we should be careful of water intoxication if patient have respiratory distress, changed mental status and electrolyte imbalance. It is seen especially in psychiatric patients so from patients and their relatives psychiatric diseases must be asked. In psychiatric patient, non-cardiogenic pulmonary edema can be associated with water intoxication. The condition may be fatal if undiagnosed and can be successfully treated. Early diagnosis is very important because of prevention of neurologic damage and lethal complications.
